# Influence of the cardiac cycle on pCASL: cardiac triggering of the end-of-labeling

**DOI:** 10.1007/s10334-017-0611-6

**Published:** 2017-03-09

**Authors:** Jasper Verbree, Matthias J. P. van Osch

**Affiliations:** Department of Radiology, C.J. Gorter Center for High Field MRI, Leiden Institute of Brain and Cognition, Leiden University Medical Center, PO-box 9600, 2300 RC Leiden, The Netherlands

**Keywords:** Perfusion magnetic resonance imaging, Cerebrovascular circulation, Pulsatile flow, Cerebral blood flow

## Abstract

**Objective:**

In arterial spin labeling (ASL), the cardiac cycle might adversely influence signal-stability by varying the amount of label created, labeling efficiency and/or transport times. Due to the long labeling duration in pseudo-Continuous ASL (pCASL), the blood labeled last contributes most to the ASLsignal. The present study investigated, using numerical simulations and in vivo experiments, the effect of the cardiac cycle on pCASL, thereby focusing on the end-of-labeling.

**Materials and methods:**

In the in vivo experiments the end-of-labeling was timed to a specific cardiac phase while a long labeling duration of >7 s was used to isolate the influence of the lastly labeled spins on ASL-signal stability.

**Results:**

Simulations showed dependence of the ASL-signal on the cardiac phase of the end-of-labeling, and that the variation in signal was more pronounced at lower heart rates. The ASL-signal variation was small (~4%), but could be effectively reduced by simulated end-of-labeling triggering. In vivo, no difference in mean CBF (*p* = 0.58) nor in CBF temporal-STD (*p* = 0.44) could be detected between triggered and non-triggered acquisitions.

**Conclusion:**

Influence of the cardiac cycle on pCASL-signal stability is small and triggering the start-of-labeling and end-of-labeling can be considered not to have practical implications to improve stability.

## Introduction

Pseudo continuous arterial spin labeling (pCASL) is the current recommended and validated non-invasive MR technique to image cerebral blood flow (CBF) [1, 2]. The pCASL sequence consists of separate label and control acquisitions which are subtracted to yield a CBF measurement. Since labeling efficiency of pCASL depends on the blood flow velocity [3], and the recommended labeling duration is approximately two times the cardiac interval, perturbations of the ASL-signal due to variation in timing of the acquisitions with respect to the cardiac cycle should be expected. Such physiological noise could potentially be reduced by synchronizing the ASL acquisitions to the cardiac cycle. Synchronizing the moment of labeling to the cardiac cycle by means of cardiac triggering has been shown to reduce signal variability over time in pulsed ASL [4, 5]. However, pulsed ASL has a much shorter labeling duration (≈20 ms) compared to pCASL (1500–2000 ms) and the generation of label is, therefore, almost instantaneous and can easily be timed to always happen at the same moment within the cardiac cycle. A similar cardiac triggering approach has previously been investigated in continuous ASL by triggering the start of the labeling. However, no improvements in signal stability were observed using this approach [6]. This might be explained by the long labeling duration that will result in asynchronous timing at the end of the labeling and the moment of imaging even when the start of the labeling was triggered.

In ASL, the magnetic labeling of blood decays with the *T*_1_ of arterial blood (at least until the label is exchanged with brain tissue after which it will decay with the *T*_1_ of tissue). Label generated later in the labeling period will be less affected by this decay and will, therefore, contribute more to the detected signal. For example, at a labeling duration of 1.8 s, label generated at the end of the labeling period contributes a factor of three more to the totally detected ASL-signal compared to label generated at the start of labeling. Therefore, the largest contribution to the ASL-signal arises from the end of the labeling period. The greatest impact of cardiac triggering on ASL-signal stability should, therefore, be expected when triggering the end-of-labeling.

Three main effects of flow velocity in the feeding arteries on pCASL signal stability can be expected. First, the amount of label generated is proportional to the volume of spins flowing through the labeling plane and, thus, on the blood flow velocity. Secondly, the labeling efficiency is known to depend on the velocity of the flowing blood. Finally, the transport time of label to the brain tissue is dependent on the blood flow velocity and will influence the relative time the label spends in the blood versus the tissue compartment. That is, faster delivery (i.e., higher flow velocity) of the label to the tissue will reduce the ASL-signal due to smaller *T*_1_ in tissue compared to the blood compartment. Therefore, in summary, both the generation and the delivery of the label are dependent on the blood flow velocity, which varies over the cardiac cycle. Both effects could have an independent, but opposite influence on the pCASL-signal stability. Furthermore, these effects are expected to have a stronger impact on the ASL-signal at the end-of-labeling than at the start of the labeling.

The aim of the present study was to investigate the influence of the cardiac cycle on the pCASL-signal by studying the influence of triggering the end-of-labeling on the pCASL-signal stability. By focusing on the end-of-labeling, the moment of the largest influence is isolated, thereby providing a worst-case scenario estimation of the influence of the cardiac cycle on the pCASL-signal and its stability. Simulations were used to assess the influence of pulsatile flow in combination with variations in heart rate on the pCASL-signal stability. Subsequently, in vivo experiments with end-of-labeling triggering were performed in normal healthy subjects to confirm the findings from these simulations. During the in vivo experiments impractically long labeling durations were employed to isolate the effect of triggering the end-of-labeling without confounding influences of variation in the total labeling duration.

## Materials and methods

### Numerical model

A numerical model was implemented in MATLAB 2016a (Mathworks, Natick, MA, USA) to investigate the effect of the cardiac cycle on the ASL-signal, by simulating both label generation and label delivery. The model was subsequently used to investigate the effect of pulsatile flow, heart rate variability and end-of-labeling triggering.

**Fig. 1:**
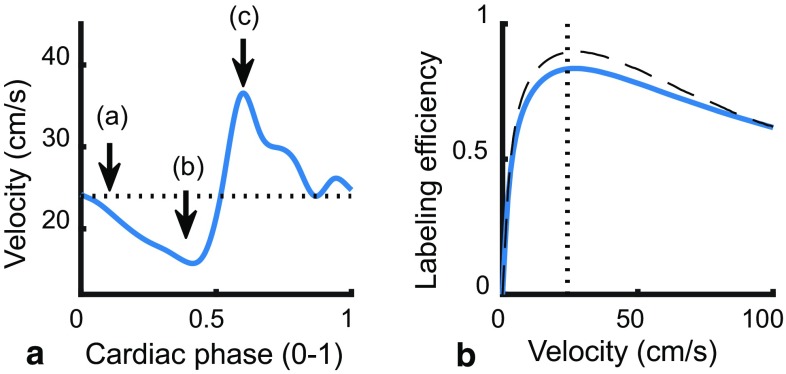
Graphical representation of **a** a typical velocity *curve* from a representative subject of the present study in the internal carotid artery, which is used as a template in the simulations and **b** the velocity dependent labeling efficiency as employed in the simulations. The labeling efficiency (*dashed line*) is adapted from the numerical model described in [10], while assuming a parabolic flow profile in the simulated artery (*blue line*). The dotted line denotes the average velocity (24 cm/s) for visual reference

The numerical model simulates the label generation in a single artery and the delivery of the labeled spins into a large tissue compartment, which acts as a sink. Fresh blood flows into the artery at the labeling plane according to a typical velocity curve as encountered in the internal carotid artery of an adult person (see Fig. 1). The arterial compartment consists of a single 300 mm long artery discretized into blocks of 0.01 mm length. Blood flow is simulated by shifting blocks, so that for blood flowing at 24 cm/s at transport time through the arterial compartment of 1250 ms is achieved. Labeling of blood is simulated by introducing blocks with inverted magnetization, while taking into account the labeling efficiency (0.85). When no labeling is applied, the blood magnetization is set to the fully relaxed magnetization (*M*_0_ = 1.0). Each iteration (1 ms) the blood is pushed into the artery. Blood at the end of the artery is subsequently pushed into the tissue compartment, which has a size equal to 250,000 blocks. Subsequently, the longitudinal (*T*_1_) magnetization of all blocks in the arterial and tissue compartments is updated according to the Bloch equations [7]:

$$M_{z} (t + {\text{d}}t) = M_{z} (t) \cdot e^{{ - {\text{d}}t/T_{1} }}  + M_{0}  \cdot (1 - e^{{ - {\text{d}}t/T_{1} }} )$$

where the time-step (d*t*) is taken as 1 ms and the *T*_1_ is set to *T*_1,blood_ (1664 ms [8]) and *T*_1,tissue_ (1105 ms, estimate for a voxel containing both gray and white matter [9]) for the arterial and tissue compartments, respectively.

The control condition of the ASL experiment is not explicitly simulated, but is assumed to be equal to the total sum of blocks making up the tissue compartment. This implies that we assume that the inflowing magnetization is only affected during the label condition and that the control condition is perfect and unaffected by the timing with respect to the cardiac cycle. The blood flow is calculated from the difference (Δ*M*) between the control condition and simulated label signal and subsequently quantified according to the consensus formula by correcting for the finite labeling duration (LBL) and post labeling delay (PLD) [1]:

$$ f = \Delta M\frac{{e^{{{\text{PLD}}/T_{1} ,{\text{blood}}}} }}{{(1 - e^{{{\text{ - LBL}}/T_{1} ,{\text{blood}}}} )}} $$

The flow, or CBF, is the parameter of interest of the simulation and will be referred to as “signal” or “ASL-signal” throughout the manuscript. The recommended timing of 1800 ms for labeling and PLD are used in the initial simulations.

Pulsatile blood flow is simulated by repeating the typical velocity curve (Fig. 1) to fill the entire duration of the simulation. To isolate the effect of flow pulsatility during labeling (or the PLD), the velocity is set, for some experiments, equal to the mean velocity (i.e., constant velocity, no pulsatility) during certain sub-parts of the ASL sequence. The heart rate was modified by altering the duration of the cardiac cycle, i.e., by linearly scaling the flow velocity curve in the temporal domain. Heart rate variability is achieved in the same way, by randomly altering the duration of each cardiac cycle. Note that the pulsatility (difference between minimum and maximum velocity) is not altered throughout the simulations. The timing of the arrival of label in the tissue compartment is characterized by the bolus arrival time (BAT) and the tail arrival time (TAT). BAT is defined as the first non-zero ASL-signal value arriving in the tissue compartment, whereas TAT is defined as the last non-zero ASL-signal value leaving the arterial compartment.

Labeling efficiency is velocity-dependent and varies between the cardiac phases. The labeling efficiency for a single velocity has been described in a numerical model [10]. However, the flow profile within the vessel varies with the distance from the vessel wall. The data from the numerical model [10] was recalculated assuming parabolic flow profile in the simulated artery (see Fig. 1b). To isolate the effects of spatially as well as temporally varying labeling efficiencies due to blood velocity differences from other influences on ASL signal stability, labeling efficiency was assumed constant for the other simulations.

The resulting ASL-signal and other variables (e.g., BAT) are expressed relative to the signal as obtained from simulations with a constant mean flow velocity as input. The relative signal (Δ*S*) is expressed as: Δ*S* = (*B* − *A*)/*A* × 100%, where *A* represents the constant and *B* is the pulsatile flow condition. The cardiac phase is defined as the normalized timing within the cardiac cycle and runs, therefore, from 0 to 1. Mean and standard deviations of the cardiac phase are calculated by taking into account that the cardiac phase “wraps around” each cardiac cycle, i.e., circular statistics [11].

### In vivo experiments

Data from eight healthy volunteers (four female) with a mean age of 30 years (range 23–52), were acquired on a Philips 3T MRI (Achieva, Philips, Best, The Netherlands). All participants were screened for MRI safety compliance, and the study protocol was approved by the Ethics Review Board of the Leiden University Medical Center. Prior written informed consent was obtained from all subjects, and the study was performed in accordance with the Helsinki protocol.

**Fig. 2:**
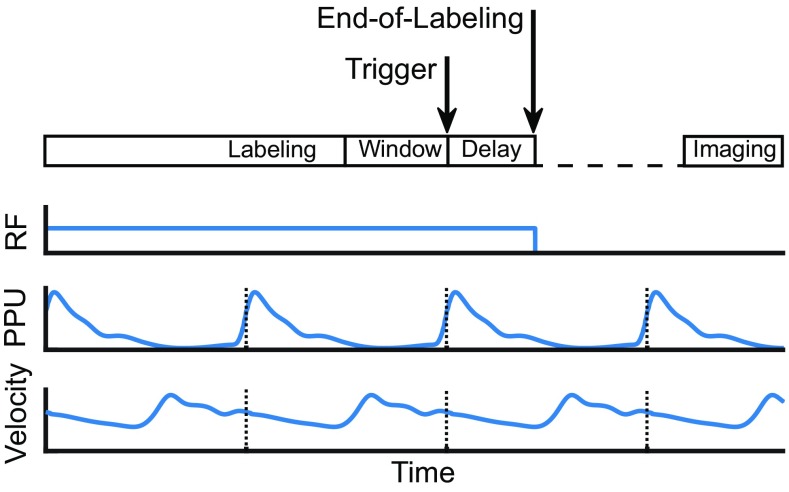
Schematic representation of the modified pCASL sequence (*top*) with respect to the labeling RF, finger pulse-oxymetry signal (PPU) and blood flow velocity at the labeling plane. “*Labeling*” indicates the minimum labeling duration (7500 ms—delay). *Arrows* indicate the cardiac trigger and moment of end-of-labeling. Note the shift between finger PPU trigger and velocity during labeling

The vendor-supplied pCASL sequence was modified to incorporate both start-of-labeling as well as end-of-labeling triggering. A graphical representation of the ASL sequence in relation to the trigger and the employed trigger-delays is depicted in Fig. 2. To stop the end-of-labeling in a certain cardiac phase, a delay parameter was set on the scanner that determines the additional labeling duration after receiving a cardiac trigger event. The delay parameter was set by the operator to three distinct cardiac phases (a–c) based upon the subject’s velocity curve, see Fig. 1. The velocity curve was obtained prior to the ASL acquisition by means of phase contrast MRA (for acquisition parameters, see Table 1). The minimum labeling duration, i.e., the moment after which the scanner will look for the next cardiac trigger, was chosen so that the expected average labeling duration was 7500 ms.

**Table 1:** Acquisition parameters

	Anatomical *T*1-weighted	Phase contrast MRA	Triggered pCASL	Non-triggered pCASL
Scan technique	3D Fast field echo	2-D Fast field echo	Multi-slice gradient echo	Multi-slice gradient echo
Acceleration type	TFE factor 179, SENSE 1.8 RL	–	SENSE factor 2.5, EPI factor 35	SENSE factor 2.5, EPI factor 35
Repetition time/echo time	9.8/4.6 ms	13/8 ms	Variable/14 ms	8200 ms (fixed)/14 ms
Flip/refocusing angle	8°/–	10°/–	90°/–	90°/–
Field of view	224 × 176 × 168 mm^3^	150 × 103 × 6 mm^3^	240 × 240 × 119 mm^3^	240 × 240 × 119 mm^3^
Acquisition voxel size	1.19 × 1.21 × 1.20 mm^3^	1.17 × 1.17 × 6.00 mm^3^	3 × 3 × 7 mm^3^	3 × 3 × 7 mm^3^
Acquisition matrix	188 × 146	128 × 128	80 × 80	80 × 80
Scan duration	2:47 min	2.5 − 4 min	Variable	7 min
Labeling duration	–	–	7000 ms (minimum)	7500 ms (fixed)
Post labeling duration	–	–	1800 ms	1800 ms
Additional parameters	Inversion delay 935 ms	Venc 200 cm/s; 2 averages; retrospective triggering; 15 cardiac phases	Set trigger delay (0 − 1000 ms)	–
TFE dur. shot/acq	1938/1757 ms			

*TFE* Turbo Field Techo, *Venc* phase-encoding velocity, *SENSE* parallel imaging technique

The triggered scan was acquired continuously, cycling through the cardiac phases (a–b–c–a–b–c…) resulting in 20 label and control images for each cardiac phase. In addition to end-of-labeling triggering, both control and label acquisitions were triggered at the start-of-labeling to exclude any residual influence of the start-of-labeling. The control condition was defined to have the same labeling duration as the preceding, end-of-labeling triggered label acquisition, this to ensure equal effects on static tissue magnetization for the control and label condition. A non-triggered reference ASL scan was acquired with the same imaging parameters and a labeling duration of 7500 ms. Total scan time was approximately 1 h, exact duration depending on the heart rate.

The post-processing of the in vivo data were performed in MATLAB v2016a (Natick, MA, US) with the SPM12 toolbox. After motion correction, the ASL-images were quantified using the consensus formula, taking into account the increase in PLD for later acquired slices (36 ms inter-slice interval with ascending slice-order, starting from 1800 ms) [1]:

$$ {\text{CBF}} = \frac{{6000 \cdot \lambda \cdot {\text{e}}^{{\frac{\text{PLD}}{{T_{{ 1 , {\text{blood}}}} }}}} }}{{2 \cdot \alpha \cdot T_{{ 1 , {\text{blood}}}} \cdot (1 - {\text{e}}^{{ - \frac{\text{lbldur}}{{T_{{ 1 , {\text{blood}}}} }}}} )}} \cdot \frac{{{\text{SI}}_{\text{control}} - {\text{SI}}_{\text{label}} }}{{M_{0} }}. $$

The quantification was performed per dynamic to correct for the varying labeling duration while triggering the end-of-labeling. The global scaling factor *M*_0_ was obtained from the maximum “pure water” CSF signal (*M*_0,csf_) in the lateral ventricles of the non-triggered scan assuming a fully relaxed signal at a repetition time of 8.2 s. The *M*_0,csf_ was corrected for the water content of arterial blood (76%) to obtain the value of fully relaxed spin in blood: *M*_0,blood_ = *M*_0,csf_ × 0.76 [12]. The average quantified CBF was subsequently calculated by employing a gray matter mask obtained from the segmentation of the *T*_1_-weighed scan. Differences between the (non-)triggered conditions were assessed using the non-parametric Friedman’s test (Matlab 2016a).

## Results

### Simulations: constant flow

**Fig. 3:**
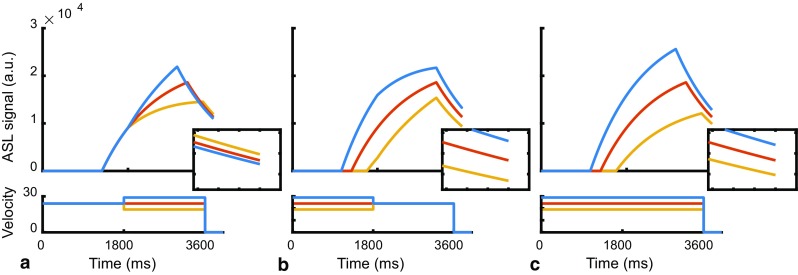
Simulated ASL-signal evolution in the tissue compartment (*top panels*) during constant flow. Constant velocity increase/decrease during: **a** PLD only; **b** labeling only; and **c** both labeling and PLD. The insets show a close-up of the signals at the moment of imaging (same scale for all insets)

The ASL-signal evolution in the tissue compartment is depicted in Fig. 3 for three constant flow conditions. Increased flow rate during the PLD (while flow during labeling remained constant) caused the tail of the labeled blood (bolus) to arrive earlier in the tissue compartment. The lower *T*_1_ in the tissue compartment with respect to the blood compartment increased the rate of signal decay. Therefore, a higher flow rate during the PLD resulted in less detected ASL-signal (see inset, Fig. 3a).

Changing the flow only during labeling had two effects (see Fig. 3b): first, the ASL-signal increased with the higher flow rate as expected, i.e., more spins are labeled; secondly, the bolus arrival time (BAT) became the shorter (horizontal axis, Fig. 3b). The second effect also leads to faster decay of the ASL-signal similar to the results for increased flow velocity during the PLD due to the shorter *T*_1_ of tissue. Comparing the signal intensity in the insets of Fig. 3, it can be appreciated that the first (i.e., flow) effect dominates over the compartmental *T*_1_-effect. Finally, increased flow during both labeling and PLD overlays previous effects (see Fig. 3c).

### Simulations: pulsatile flow

**Fig. 4:**
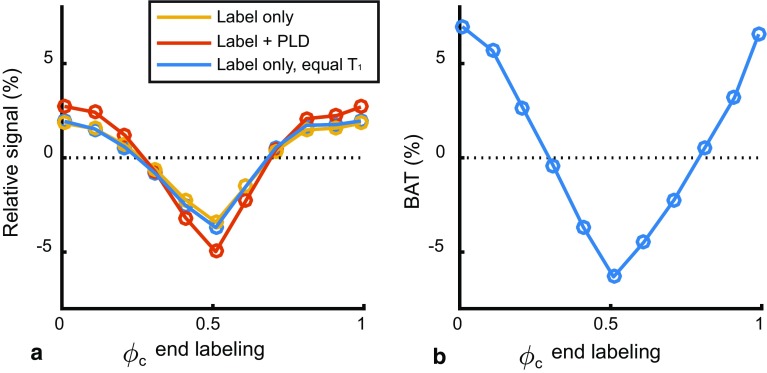
Simulated pulsatile flow of two cardiac cycles during labeling (R–R interval 900 ms = 67 bpm). **a** ASL-signal of pulsatile flow during label only (*yellow*), label only and equal *T*_1_ for blood and tissue compartments (*blue*) and pulsatile flow during labeling and PLD (*red*). **d** Bolus arrival time (BAT). BAT *curve* was equal for all three conditions; therefore, a *single curve* is shown. All signals are normalized to the constant flow condition and aligned to the cardiac phase at the end-of-labeling

The effect of pulsatile flow on the signal inflow in the tissue compartment is depicted in Fig. 4. The labeling duration (1800 ms) was set exactly equal to two cardiac cycles (R–R interval 900 ms) and the velocity of blood during the PLD was set to the average velocity over one cardiac cycle. Therefore, the volume of blood that is labeled was constant for each simulation and, thus, independent of the cardiac phase of the start-of-labeling.

The pulsatile flow can be seen to influence the ASL-signal relative to the simulation with constant flow (Fig. 4a), even while the volume of label created was equal and constant. Therefore, differences in the exact timings when label is created (start- versus the end of the labeling) appear to influence the detected signal even after accounting for loss of label due to longitudinal relaxation. Changes in the bolus arrival time (Fig. 4b) illustrate the variation in timing of label delivery and the influence of compartmental *T*_1_ differences. Setting the same *T*_1_ for both compartments resulted in a very similar signal profile (Fig. 4a, yellow curve). Finally, adding pulsatile flow during the PLD (Fig. 4a, red curve) resulted in a slightly stronger influence on the timing of delivery (i.e., of the bolus tail), which induced only small differences in signal fluctuations.

### Simulations: heart rate

In normal physiological conditions the heart rate will not exactly match the labeling duration and, consequently, the volume of labeled blood will vary, depending on the cardiac phase of the start- and, thus, also the end-of-labeling. Multiple fixed heart rates between 40 and 90 bpm were simulated ,and the phase of the start-of-labeling was varied to cover the entire cardiac cycle. First, the effect of pulsatile flow during labeling was investigated (while flow was constant during the PLD).

**Fig. 5:**
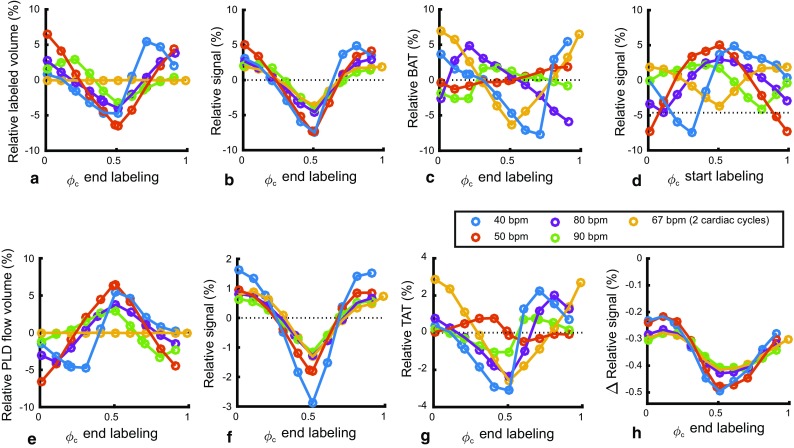
Simulated multiple heart rates with pulsatile flow during labeling and PLD. **a**–**d** Pulsatile flow during labeling, while PLD flow was constant. The special condition of exactly two cardiac cycles in the labeling period is shown in *yellow* (67 bpm), i.e., the situation of Fig. 4. **e**–**g** Simulation of fixed heart rates with pulsatile flow rate during PLD only. Flow during labeling was constant. TAT: tail arrival time of the bolus, with a positive value meaning later arrival in tissue compartment. **d** Same relative signal as in **b**, only then aligned to the start of the labeling. All values are normalized to the constant flow condition. **h** Difference in simulated relative signal between constant and velocity dependent labeling efficiency. Difference values are expressed in percentage points

The volume of labeled spins (i.e., the number of spins labeled, which should not be confused with the detected ASL-signal at which point the label will have decayed considerably) can be seen to depend on the cardiac phase when labeling stopped (Fig. 5a) [except for the case when labeling equals exactly two cardiac cycles when the labeled volume was, again, constant (Fig. 5a, 67 bpm yellow)]. The relative signal variation over the cardiac cycle (min–max range) was notably higher at lower heart rates (Fig. 5b). Although visually there is a strong resemblance between the shape of labeled volume and the ASL-signal, the special case of constant labeling volume (Fig. 5a, 67 bpm yellow) illustrates that there is a similar amount of signal variation even when there is no variation in labeled volume. Moreover, the curves in Fig. 5b are all synchronized with respect to the end of-labeling, whereas after sorting them versus the start of labeling (Fig. 5d) this coherence is lost. Therefore, the signal variation in Fig. 5b shows an effect of the end-of-labeling rather than an effect of labeled volume or start-of-labeling.

The effect of pulsatile flow during the PLD (while keeping flow constant during labeling) on the relative signal showed a very similar pattern as the relative signal due to pulsatile flow during labeling (compare Fig. 5a–c with Fig. 5e–g). The effect of label generation and delivery will, therefore, reinforce each other, independent of heart rate.

### Simulations: labeling efficiency

Until this moment labeling efficiency was assumed constant over the cardiac cycle;, however, it is known to be dependent on the velocity of the blood [10, 13]. Velocity-dependent labeling efficiency (Fig. 1) was, therefore, added to the simulations with pulsatile flow during labeling and PLD. Similar curves were obtained as with constant labeling efficiency: the difference between constant and velocity dependent labeling efficiency was only −0.35 ± 0.08 percentage point (95%-confidence interval: −0.37, −0.33%), as shown graphically in Fig. 5h. The influence of labeling efficiency was more pronounced at higher heart rates.

### Simulations: labeling duration

**Fig. 6:**
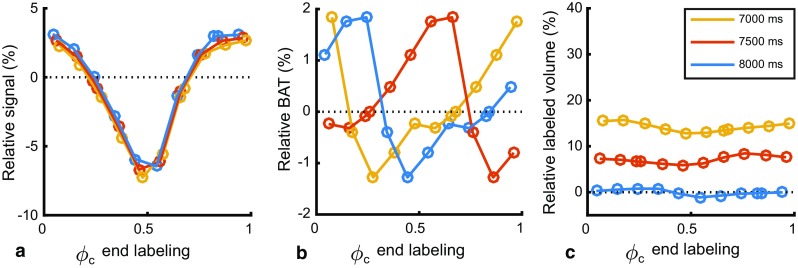
Simulated effect of additional labeling duration on: **a** relative ASL-signal; **b** bolus arrival time and **c** labeled volume. Pulsatile flow was simulated during labeling and PLD at a fixed heart rate of 50 bpm. All values are normalized to the constant flow condition with a labeling duration of 7000 ms

At a very long labeling duration of 7 s, increasing the labeling duration by 1 s had no effect on the relative signal within the precision of the simulation (Fig. 6a). As the cardiac phase of end-of-labeling shifts with increasing labeling duration, the BAT can be seen to shift as well (Fig. 6b). The relative labeled volume increases with labeling duration as expected (Fig. 6c).

### Simulations: triggering the end-of-labeling

The bridge to the in vivo experiments was made by simulating the end-of-labeling triggering using the same parameters as during the in vivo experiments. After labeling for minimally 7 s, labeling was continued for an additional labeling period after a trigger; this was dubbed “trigger delay” (see “In-vivo experiment methods” section for details). The normal physiological situation was simulated by adding heart rate variability (HRV) to the cardiac cycle. For this simulation, a pulsatile flow profile with a HRV of 100 ms was generated at two mean heart rates of 40 and 90 bpm as two extreme cases. Due to the stochastic nature of the HRV, each trigger-delay was simulated 33 times. The start-of-labeling was also triggered similarly as done in the in vivo experiment. The non-triggered case was simulated 33 times with a mean labeling duration of 7500 ms.

**Fig. 7:**
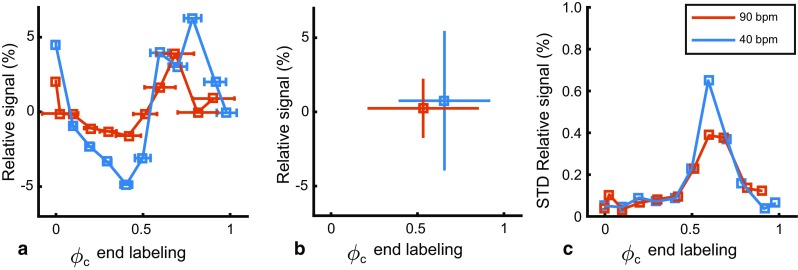
Simulated end-of-labeling triggering at an average heart rate of 40 bpm (*blue*) and 90 bpm (*red*) with a heart rate variability of 100 ms. **a** Relative ASL-signal with triggering. **b** Relative ASL-signal without triggering (random selection of 33 data points from **a**). **c** Standard deviation of data in **a**. All values are normalized to the constant flow condition. Error bars indicate the standard deviation

From Fig. 7a it can be appreciated that HRV introduces uncertainty in the determination of the exact phase of the end-of-labeling (i.e., variation in the *x* axis). A similar pattern in relative ASL-signal as in Fig. 5b can be observed. As the variation in ASL-signal due to labeled volume is negligible at these long labeling durations, this is mostly the effect of the end-of-labeling phase. The variation in signal is, again, more pronounced at the lower heart rate both for the triggered (Fig. 7a) as well as for the non-triggered (Fig. 7b) case. The standard deviation of the non-triggered ASL-signal averaged over the cardiac cycle was 4.7 and 2.0%, for 40 and 90 bpm, respectively (see Fig. 7b). The standard deviations along the *y* axis in Fig. 7a were plotted separately in Fig. 7c. A strong reduction in standard deviation can be observed at any end-of-labeling phase when triggering (see Fig. 7c) compared to the non-triggered (see Fig. 7b).

### In vivo experiments

Physiological parameters as measured during the in vivo triggered and non-triggered scans are provided in Table 2. Heart rate (*p* = 0.31) and mean (*p* = 0.65) and standard deviation (*p* = 0.63) of the labeling duration did not differ significantly between scans or trigger condition. When comparing the phase in the cardiac cycle that we aimed for by setting the post-trigger delay with the actual phase at the end-of-labeling, a close correspondence is found (Table 2). The combination of triggering and set delay showed little variation around the intended cardiac phase [5% at longest tested delay (c)].

**Table 2:** Physiological values for the in vivo experiment

	(a)	(b)	(c)	NT	*p* value
Heart rate (bpm)	59 ± 8	59 ± 8	59 ± 8	62 ± 11	0.08
Label duration (ms)	7517 ± 97	7492 ± 140	7475 ± 123	7455 ± 7	0.65
Label duration tSTD (ms)	311 ± 95	284 ± 66	324 ± 74		0.63
Set delay (ms)	70 ± 0	418 ± 78	576 ± 82		
Cardiac phase (0–1)					
End-of-labeling	0.07 ± 0.01	0.40 ± 0.06	0.55 ± 0.07	0.70 ± 0.20	
End-of-labeling tSTD	0.01 ± 0.00	0.03 ± 0.01	0.05 ± 0.02	0.20 ± 0.01	
Imaging	0.71 ± 0.19	0.03 ± 0.21	0.83 ± 0.22	0.68 ± 0.17	0.78
Imaging tSTD	0.11 ± 0.02	0.13 ± 0.02	0.14 ± 0.03	0.20 ± 0.01	0.54

Values are mean ± std. tSTD is the temporal standard deviation over 20 dynamics. *p* value indicates the repeated-measures ANOVA comparison between the four conditions

**Fig. 8:**
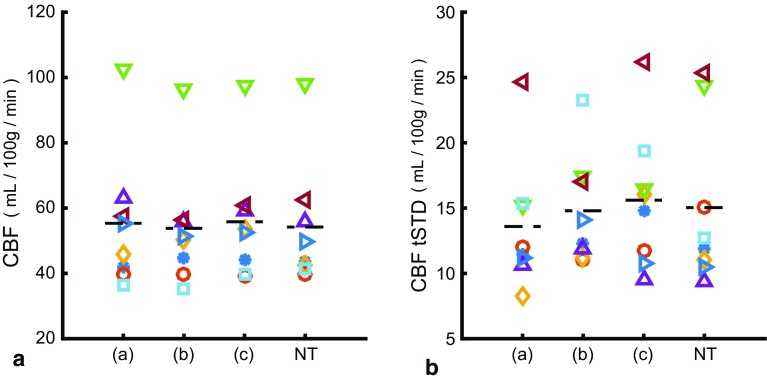
In-vivo measurements of mean gray matter CBF and tSTD of the triggered and non-triggered scans. tSTD is the temporal standard deviation over 20 dynamics. The moment of end-of-labeling in the cardiac cycle **a**–**c** was defined by a set delay and cardiac triggering of the end-of-labeling. The cardiac phase of end-of-labeling varied randomly during the non-triggered scan (NT). Removing the potential outlier (*green triangles*) reduced the mean CBF to 48.5 ± 8.4 ml/100 g/min, without effect on statistical inferences

The mean gray matter CBF was 55.34 ± 21.21, 53.81 ± 18.85 and 55.80 ± 18.78 ml/100 g/min for the triggered conditions a,b, and c and 54.19 ± 19.37 ml/100 g/min for the non-triggered scan. No difference in mean CBF between the four conditions could be detected (*p* = 0.58, Friedman’s test), see Fig. 8a. The temporal standard deviation of the CBF (13.59 ± 5.04, 14.79 ± 4.23, 15.60 ± 5.38 and 15.04 ± 6.31 ml/100 g/min) was comparable between the four conditions (*p* = 0.44), see Fig. 8b.

## Discussion

The present study investigated the effect of the cardiac cycle on pCASL and especially focused on the effect of end-of-labeling triggering on ASL-signal stability. Simulations showed a clear, although small (on the order of 4% of the ASL-signal) influence of flow pulsations on the ASL-signal, even when the labeling duration spanned multiple cardiac cycles. The timing of the pulsatile flow was shown to influence both the generation- and delivery of the label, and the resulting effect on the ASL-signal was most associated with the end-of-labeling phase. However, the in vivo experiments could not detect a difference in mean ASL-signal or temporal ASL-signal stability between the cardiac phases of the end-of-labeling triggering and/or with the non-triggered scan.

This study aimed to investigate the influence of the cardiac cycle on pCASL measurements of CBF, particularly during the end-of-labeling, since that timing will affect the ASL-signal stability most. To isolate the influence of cardiac cycle during the end-of-triggering on ASL-signal stability, an impractical long labeling duration was employed and combined with end-of-labeling triggering: by using these very long labeling durations (>7 s) in the in vivo experiment, the occurrence of additional labeling of, e.g., 1 s had little influence on the overall signal as was confirmed by the simulations (see Fig. 6). When translating this to practical scan properties, triggering the end-of-labeling could be a candidate approach to minimize signal instabilities due to the cardiac cycle, although it might introduce more unwanted effects than solving when employed with commonly employed labeling durations. For instance, at a typical heart rate of 60 bpm, the additional labeling duration could vary up to 1 s; therefore, the ASL-signal change due to the volume of additionally generated label at 2.8 s compared to 1.8 s labeling, could overshadow the potential effect of increased signal stability due to end-of-labeling triggering, while greatly complicating the consistency of CBF quantification. To provide equal quantitative CBF-estimation over such a wide range of labeling durations, it would be crucial to have an accurate estimate of dispersion of the bolus of labeled spins [4] and the exact exchange time of the label from the intra- to the extravascular compartment [14], which are both difficult to estimate in an individual subject. Therefore, the present study aimed first to study how large the effect of the cardiac cycle is on the signal stability, focusing thereby on the potential benefit and properties of end-of-labeling triggering before moving on to solving all the implementation issues that would be encountered when using such an approach in practical clinical applications. The simulation and in vivo results suggest; however, that implementing end-of-labeling triggering does not provide important improvements in signal stability for most pCASL applications. Therefore, it was not deemed useful to solve the aforementioned implementation challenges. It should be noted that during the in vivo experiments, only the label condition was end-of-labeling triggered, whereas the following control condition was acquired with the same ‘labeling’ duration. Hence, the repetition time (TR) was the same for each label and control pair, thereby avoiding that magnetization differences would affect pair-wise subtractions. Even when controlling for these confounding effects, no improvement in signal stability could be proven in the present in vivo study when using end-of-labeling triggering.

The simulations in the present study showed that signal fluctuations due to cardiac pulsations range between −8 and +5%. Previous work with simulations of continuous ASL showed a similar ±5% range in signal variation for a labeling duration of 1500 ms (see Fig. 7 of Ref. [4]). However, their simulations (convolution of the velocity curve with the Buxton model) only took the effects into account that occurred while generating the label. Variation in the transport time of the label due to the cardiac cycle was not part of that continuous ASL study, although it was included in other studies, which investigated pulsed ASL [5, 15–17]. Both modeling and in vivo pulsed ASL data showed earlier arrival of the label with the systolic phase compared to the diastolic phase [5, 15–17]. The simulations of the present study extend this concept to pCASL and showed that the delivery of label enhanced the signal fluctuations of labeling generation. In other words, the changes in transport times during the PLD due to cardiac fluctuations augment the ASL-signal increases and decreases caused by flow pulsations during labeling. Hence, the range in signal variations of the present simulations is slightly larger than earlier predicted in literature. Moreover, this study is the first to show that these signal fluctuations are largely dependent on the phase of end-of-labeling and not with the start-of-labeling. Finally, the influence of the cardiac cycle on the ASL-signal due to the changed transport times and the differences in the amount of generated label, were both greater than changes due to the velocity dependency of the labeling efficiency, which is often cited as a major concern in pCASL.

In the simulations we quoted the influence of the cardiac cycle on ASL-signal by stating the observed signal range (−8 to +5%) to illustrate the maximum range of expected ASL-signal intensities; however, it could also have been expressed as temporal (tSTD), which has the advantage that it can be measured reliably in vivo. The tSTD of the simulated untriggered case was 4% at the lowest heart rate, which in the simulations reduced to 0.08% tSTD when end-of-labeling triggering was employed. The relative small effect of 4% shows that influence of the cardiac cycle is probably only a minor factor of observed ASL-signal fluctuations. Therefore, other physiological factors, such as respiration, might play a bigger role in pCASL [6, 18].

In the present study, the average coefficient of variation measured in the non-triggered scan coefficient of variation was 28 ± 11%, and; therefore, the number of subjects in the present study can be considered to be too small to be able to show a 4% difference in temporal STD. However, the number of participants was chosen on the ability to prove significant impact on single subject pCASL experiments. When the effect of end-of-triggering could not be proven in a group of eight subjects, it is clear that the total effect of cardiac pulsations can be considered minor in pCASL. Taken together, the simulations and in vivo experiments suggest that the effect of cardiac pulsations is small and triggering the end-of-labeling (and therefore also the start-of-triggering) provides no consistent improvement in ASL-signal stability on a per-subject basis.

Other methods, besides cardiac triggering, have also been proposed to reduce noise in ASL. Background suppression pulses have been shown to improve the general ASL-signal stability independent of cardiac pulsations [6]. Background suppression is particularly effective when using a read-out option with a single excitation pulse such as 3D-GRACE or 3D FSE stack-of-spirals [1]. Application of background suppression in the present study was not included, because of the high implementation complexity due to the varying TR of each label-control pair. Physiological noise reduction can also be achieved during post-processing, for example, by using RETROICOR [6, 18]. By design, RETROICOR includes the cardiac and respiratory regressor determined at the moment of imaging into account, whereas the influence of the cardiac cycle at the start-of-labeling or end-of-labeling was not considered [4, 6, 18]. Application of RETROICOR in the present study proved to be of limited value due to the low number of dynamics for each condition (20 label-control pairs) and was, therefore, omitted from the final analysis. Although these post-processing techniques provide a consistent way to reduce (physiological) noise from ASL, it would be beneficial when these noise-sources would be suppressed during the acquisition of the signal. As an extreme variant of triggered ASL, we considered and tested “triple triggered” pCASL (triggering the start- and the end-of-labeling as well as the moment of imaging), which would appear to be more in line with physiology than the independent chosen values as currently employed. Triple triggered ASL leads, however, to ASL-signals that are mainly proportional to the duration cardiac cycle, and should, therefore, be considered to be not very informative.

The present study modeled the arterial compartment as a rigid vessel without compliance. Arterial compliance is an important mechanical property that gradually reduces the flow pulsatility along the arterial tree toward the tissue compartment. Generation of label at the proximal end of the artery is governed by the typical flow curve (Fig. 1), which already included the compliance of the arterial tree. The delivery of the label to the tissue was shown in the present study to play a minor role compared to generation. Compliance decreases the flow pulsatility during the delivery phase, which would in turn reduce variations in delivery time and, thus, increase overall signal variability. Therefore, the assumption of a rigid arterial compartment can be considered to provide a ‘worst-case’ scenario for the influence of flow pulsations.

The present study used the build-in finger pulse-oxy triggering of the MRI-scanner to synchronize the end-of-labeling with the cardiac cycle, which always lags behind in time compared to ECG-triggering. The present simulations suggest that the optimal moment of triggering lies at the end-systolic phase, which corresponds to the finger pulse-oxy trigger with a delay of 0 ms. Although ECG triggering would allow more flexibility in choosing end-of-labeling phases within the systole, the practical application of finger triggering in the present study can be expected not to have influenced the accuracy (see Table 2) of the end-of-labeling triggering.

## Conclusions

In summary, simulations showed that flow pulsations can influence the ASL-signal by approximately 4%, and that especially the cardiac cycle at the end-of-labeling is of importance. However, in vivo experiments provided no difference in mean signal and/or signal stability. Combined with earlier findings concerning cardiac triggering, neither start-of-labeling nor end-of-labeling triggering improves signal stability significantly, suggesting that cardiac triggering is not very beneficial for pCASL. Post-processing methods that include cardiac and/or other physiological regressors (including the timing at the end-of-labeling) could be considered to correct most of the remaining minor influences of the cardiac cycle on pCASL stability.
